# Osteoblastic *Wls* Ablation Protects Mice from Total Body Irradiation-Induced Impairments in Hematopoiesis and Bone Marrow Microenvironment

**DOI:** 10.14336/AD.2022.1026

**Published:** 2023-06-01

**Authors:** Hyun-Jaung Sim, Han-Sol So, Sher Bahadur Poudel, Govinda Bhattarai, Eui-Sic Cho, Jeong-Chae Lee, Sung-Ho Kook

**Affiliations:** ^1^Department of Bioactive Material Sciences, Research Center of Bioactive Materials, Jeonbuk National University, Jeonju 54896, South Korea.; ^2^Cluster for Craniofacial Development and Regeneration Research, Jeonbuk National University, Jeonju 54896, South Korea.; ^3^Department of Basic Science & Craniofacial Biology, College of Dentistry, New York University, New York, NY 10010, USA.; ^4^Institute of Oral Biosciences and School of Dentistry, Jeonbuk National University, Jeonju 54896, South Korea.

**Keywords:** Wntless, total body irradiation, conditional knockout, bone marrow-conserved stem cells, hematopoietic radioprotection, long-term repopulation

## Abstract

Ionizing irradiation (IR) causes bone marrow (BM) injury, with senescence and impaired self-renewal of hematopoietic stem cells (HSCs), and inhibiting Wnt signaling could enhance hematopoietic regeneration and survival against IR stress. However, the underlying mechanisms by which a Wnt signaling blockade modulates IR-mediated damage of BM HSCs and mesenchymal stem cells (MSCs) are not yet completely understood. We investigated the effects of osteoblastic Wntless (*Wls*) depletion on total body irradiation (TBI, 5 Gy)-induced impairments in hematopoietic development, MSC function, and the BM microenvironment using conditional *Wls* knockout mutant mice (*Col-Cre*;*Wls^fl/fl^*) and their littermate controls (*Wls^fl/fl^*). Osteoblastic *Wls* ablation itself did not dysregulate BM frequency or hematopoietic development at a young age. Exposure to TBI at 4 weeks of age induced severe oxidative stress and senescence in the BM HSCs of *Wls^fl/fl^* mice but not in those of the *Col-Cre*;*Wls^fl/fl^* mice. TBI-exposed *Wls^fl/fl^* mice exhibited greater impairments in hematopoietic development, colony formation, and long-term repopulation than TBI-exposed *Col-Cre*;*Wls^fl/fl^* mice. Transplantation with BM HSCs or whole BM cells derived from the mutant, but not *Wls^fl/fl^* mice, protected against HSC senescence and hematopoietic skewing toward myeloid cells and enhanced survival in recipients of lethal TBI (10 Gy). Unlike the *Wls^fl/fl^* mice, the *Col-Cre*;*Wls^fl/fl^* mice also showed radioprotection against TBI-mediated MSC senescence, bone mass loss, and delayed body growth. Our results indicate that osteoblastic *Wls* ablation renders BM-conserved stem cells resistant to TBI-mediated oxidative injuries. Overall, our findings show that inhibiting osteoblastic Wnt signaling promotes hematopoietic radioprotection and regeneration.

## INTRODUCTION

Wnt ligands are involved in various signaling pathways that are essential for development, maintenance, and homeostasis of adult tissues [1,2]. Wnt signaling plays important roles in the maintenance, self-renewal, and functional integrities of hematopoietic stem cells (HSCs) and mesenchymal stem cells (MSCs) in bone marrow (BM) niches [3-5]. The secretion of Wnt ligands is tightly regulated by Wntless (*Wls*), which encodes the receptor for Wnt ligands in Wnt-secreting cells. Therefore, *Wls* ablation impairs Wnt ligand secretion and causes various Wnt-loss-of-function phenotypes, depending on the target cells in which *Wls* is deleted [6-8]. *Wls* also functions in mature osteoblasts to regulate bone mass accrual [9,10].

Exposure to ionizing radiation (IR) causes long-term residual BM injury, with senescence and impaired self-renewal of BM HSCs [11,12]. IR-mediated damage to HSCs is closely associated with the cellular accumulation of reactive oxygen species (ROS) and resultant oxidative damage [13-15]. Similarly, our previous findings indicated that total body irradiation (TBI) induces mitochondrial ROS accumulation and senescence in BM HSCs and reduces the colony forming and reconstituting capacities of BM-conserved stem-like cells [16]. Our recent study also highlighted that osteoblastic deletion of *Wls* causes tremendous oxidative stress and senescence in BM MSCs and an impaired BM microenvironment at even a young age and gradually leads to hematopoietic disruption in old age [10]. All these findings suggest that, similar to the IR-mediated phenotypes, osteoblastic *Wls* depletion induces age-related functional damage in BM HSCs and MSCs by inducing senescence and ROS accumulation. Cellular phenotypes caused by osteoblastic *Wls* ablation might also support the hypothesis that blocking Wnt signaling enhances cellular susceptibility to oxidative stress-mediated DNA damage and apoptosis. Indeed, the upregulation of Wnt/β-catenin signaling not only enhances radioresistance among mammary progenitor cells [17] but also suppresses cellular DNA damage and apoptosis [18,19]. Those reports indicate that cellular radioresistance is ameliorated via the downregulation of the Wnt signaling pathway. However, Zhang et al. reported an opposite finding, that activation of Wnt/β-catenin signaling induces intracellular ROS accumulation and the senescence of MSCs [20]. Moreover, supplemental Dickkopf-related protein 1 (DKK1), a Wnt inhibitor, promoted hematopoietic regeneration and differentiation *in vitro* and *in vivo*, whereas pharmacological inhibition or genetic deletion of DKK1 in osterix-expressing BM cells abrogated that hematopoietic radioprotection [21].

Although numerous studies have shown that Wnt signaling or Wnt signaling-associated molecules play important roles in BM niche cells, whether Wnt signaling inhibition enhances hematopoietic recovery under IR stress and if so, by what cellular mechanisms remain unclear. In this work, we investigated how blocking osteoblastic Wnt secretion via conditional *Wls* knockout in type I collagen (*2.3 kb*-*Col1a1*)-expressing cells modulated BM maintenance, function, and repopulation of HSCs and MSCs following TBI. This is because the ablation of *Wls* by *Col2.3*-*Cre* avoids functional redundancy among Wnts, severe osteopenia, and premature lethality [5,6,22], whereas it allows exploration on the roles of long-term deficiency of osteoblastic Wnts on bone homeostasis and function of BM-conserved stem cells [10,23,24]. Our findings demonstrate that osteoblastic *Wls* deletion renders BM HSCs resistant to TBI-mediated senescence. Our results also highlight that osteoblastic *Wls* ablation protects against TBI-mediated impairments in colony formation, long-term repopulation, and self-renewal among BM HSCs and improves the survival rate of lethally irradiated mice. Overall, this study shows that *Wls* ablation in *Col1a1*-expressing cells prevents TBI-mediated augmentation of senescent MSCs and maintains bone mass accrual under IR stress.

## MATERIALS AND METHODS

### Study approval, animals, and genotyping

This study strictly followed the Guide for Animal Care and Use of Jeonbuk National University. Experimental procedures were approved by the University Committee on Ethics in the Care and Use of Laboratory Animals. All mice were housed and bred in the Animal Faculty of Dental Research (LML-18-620) at the Jeonbuk National University School of Dentistry. *Wls*-floxed allele (*Wls^fl/fl^*) mice, *Col2.3-Cre*, and ROSA26 (*R26R*) reporter mice were purchased from Jackson Laboratory [23,25,26]. The *Col2.3-Cre* mice were crossed with the *Wls^fl/fl^* mice to generate *Col-Cre;Wls^fl/fl^* mice, and *Wls^fl/fl^* mice were used as littermate controls. C57BL/6 (B6) CD45.1 and CD45.2 congenic mice (3-weeks-old; Orient Bio, Seoul, South Korea) were used for the competitive and noncompetitive transplantation assays, as well as for the survival assay. Genotyping of the transgenic mice was conducted by allele-specific PCR using *Wls^fl^*-, *R26R*-, or *Col2.3-Cre*-specific oligonucleotide primers, as described previously [10]. After genotyping, mice from different cages, but within the same experimental group, were randomly selected for TBI.

### Chemicals and laboratory equipment

Unless specified otherwise, chemicals and laboratory consumables were purchased from Sigma-Aldrich Co. LLC (St. Louis, MI, USA) and Falcon Labware (BD Biosciences, Franklin Lakes, NJ, USA), respectively.

### X-galactosidase (X-gal) staining

To histologically compare the spatial patterns of *Col2.3-Cre* activity in the BM and spleen, *Cre*-mice were crossed with *R26R* mice expressing the *LacZ* gene in a *Cre*-mediated excision of a floxed cassette [26]. At 4 weeks of age, gene activity was assessed in the BM and spleens of the *Col2.3;R26R* mice via X-gal staining. All procedures for X-gal staining followed methods described previously [10]. Briefly, femur and spleen tissues were dissected, fixed in 4% paraformaldehyde, washed twice with phosphate-buffered saline (PBS), and then soaked overnight in 30% sucrose/PBS at 4°C. The tissues were embedded in Tissue-Tek O.C.T. Compound (25608-930; Sakura Finetek USA, Inc., Torrance, CA, USA), sectioned using a cryostat into a thickness of 18 μm, and mounted on gelatin-coated slides. After a serial incubation in fixation and detergent solutions, the sections were further incubated with X-gal staining solution at 37°C for 3 h followed by counterstaining with nuclear fast red solution in a darkroom.

### TBI exposure

Four-week-old *Col-Cre;Wls^fl/fl^* and *Wls^fl/fl^* mice were exposed to sub-lethal TBI (5 Gy) with γ-rays on a rotating platform (Model 109-85 series- JL Shepherd & Associates, San Fernando, CA, USA) by regulating the dosage time using the radioactive half-life of γ-rays. After various days of irradiation, the TBI-exposed and -unexposed mutant and *Wls^fl/fl^* mice were processed for further experiments: flow cytometry, colony forming assay, quantitative real time-polymerase chain reaction (qRT-PCR), enzyme-linked immunosorbent assay (ELISA), immunohistochemistry (IHC), blood cell counting, osteogenic differentiation assay, and micro-computerized tomographic (μCT) analyses. In the transplantation assay, the conditioned recipient mice were exposed to lethal TBI (10 Gy) using the same IR system.

### Flow cytometry

The numbers and phenotypes of BM- or spleen-conserved stem cells in the *Col-Cre;Wls^fl/fl^* and *Wls^fl/fl^* mice were determined using multicolor flow cytometry (BD Aria, BD, Bioscience Franklin Lakes, NJ, USA) installed in the Center for University-Wide Research Facilities (CURF) at Jeonbuk National University. Here BM cells were harvested at early times (12, 24, and 72 h) and 4 weeks after TBI exposure by flushing the femur and tibia with PBS using a syringe, without crushing bones or treating with collagenase. Cells from spleen tissue were collected from these mice at the same time followed by treatment with red blood cell (RBC) lysis buffer for 15 min on ice. After washing with PBS, cell populations were sequentially gated and analyzed using FlowJo software and methods described previously [10,27]. Unless specified otherwise, antibodies were purchased from BD Biosciences. The phenotypes of BM- and spleen-derived Lin^-^Sca-1^+^c-Kit^+^ (LSK), CD150^+^CD48^-^LSK (HSCs), and Lin^-^Sca-1^-^c-Kit^+^ cells (hematopoietic progenitor cells, HPCs) were identified using the lineage markers phycoerythrin (PE)-Cy7-conjugated anti-CD3 (#552774), anti-CD4, anti-CD8, anti-CD45R (B220; #552772), anti-CD11b (#552850), anti-Gr-1 (#552958), and anti-TER-119 (#557853); PE- (#553108) or fluorescein isothiocyanate (FITC)-conjugated anti-stromal cell-derived factor 1 (Sca-1; #557405); allo-phycocyanin (APC)-conjugated anti-c-kit (#553356); PerCP/Cy5.5-conjuated anti-CD150 (#46-1502; eBioscience, Waltham, MA, USA); and APC-Cy7-conjugated anti-CD48 (#561826). The phenotypes of BM-derived HPCs, granulocyte-monocyte progenitor (GMP), common myeloid progenitor (CMP), megakaryocyte-erythroid precursor (MEP), and common lymphoid progenitor (CLP) cells, were characterized using PE-conjugated anti-FcR, PerCP/Cy5.5-conjuated anti-CD34, and PE-conjugated anti-IL-7R. Populations of Lin^-^Sca-1^+^CD29^+^CD105^+^ cells were phenotypically identified as BM-derived MSCs using the same APC-Cy7-conjugated Sca-1, PE- or FITC-conjugated CD29, and APC-conjugated CD105 antibodies. The numbers of circulating granulocytes (Gr-1^+^), monocytes (CD11b^+^), T cells (CD3^+^), and B cells (B220^+^) in peripheral blood (PB) were phenotypically defined using PE-Cy-, FITC-, APC-, and PE-conjugated antibodies, respectively. A flow cytometric analysis was also performed to determine the oxidative stress- or senescence-related phenotypes of HSCs and MSCs derived from TBI-exposed and -unexposed mutant and *Wls^fl/fl^* mice. To that end, the mitochondrial superoxide anion level and senescence-associated β-galactosidase (SA-β-gal) activity were assessed using MitoSox Red (#M36008; Invitrogen, Carlsbad, CA, USA) and C_12_FDG (#I2904; Molecular Probes, Eugene, OR, USA), respectively. In addition, the level of p16^INK4a^ in the BM HSCs and MSCs was determined flow cytometrically using Alexa Fluor 488-conjugated antibody (sc-56330 AF488; Santa Cruz Biotechnology, Santa Cruz, CA, USA).

### Transplantation assay

We investigated donor cell-derived repopulation capacity using the CD45.1/CD45.2 congenic systems. Briefly, the recipient mice were exposed to lethal TBI (10 Gy) 12-24 h prior to transplantation. The first conditioned recipients (1° TP recipients) received an HSC transplant by tail vein injection of 10^3^ cells taken 4 weeks post-TBI from *Wls^fl/fl^* (CD45.2) or *Col-Cre;Wls^fl/fl^* (CD45.2) mice exposed to sub-lethal TBI, in combination with HSCs (10^3^ cells) derived from competitor mice (CD45.1). BM cells (2×10^6^ cells) isolated from the 1° TP recipients were noncompetitively transplanted into the second recipients (2° TP), which was followed by the same transplantation procedure into the third recipients (3° TP) with a 5-month interval after each transplantation. The long-term repopulating potential of donor cells in the recipients was evaluated after each transplantation. The engrafted numbers and populations of C_12_FDG- and p16^INK4a^-positive HSCs in the BM of the 1° TP recipients were analyzed by flow cytometry 5 months post-transplantation. The populations of myeloid lineages (Gr-1^+^ and CD11b^+^ cells) and lymphoid lineages (CD3^+^ and B220^+^ cells) in the PB of the 1° TP recipients were determined by flow cytometry 3 months post-transplantation. In addition, the ability of the donor cells to protect the recipients against lethal irradiation was evaluated by transplanting HSCs (10^4^ CD45.2-expressing cells) sorted from the BM of the 1° TP recipients into conditioned recipient mice. The survival of the recipient animals was monitored for up to 12 months post-transplantation.

### Cell culture and colony forming unit (CFU) assay

We analyzed the effects of osteoblastic *Wls* deficiency on TBI-mediated changes in the HPC potential to form colonies. Briefly, BM HPCs were isolated from *Wls^fl/fl^* and *Col-Cre*;*Wls^fl/fl^* mice 4 weeks post-TBI and then cultured in 35-mm culture dishes (2 × 10^4^ cells/dish) with MethoCult^®^ GF M3434 medium (STEMCELL Technologies). After 12 days of incubation, the numbers of CFU-granulocytes/macrophages (CFU-GMs), burst forming unit-erythrocytes (BFU-Es), and CFU-granulocyte/erythroid/macrophage/megakaryocyte colonies (CFU-GEMMs) were counted using standard criteria. Alternatively, whole BM cells were harvested from the tibia and femurs of TBI-exposed and -unexposed mutant and *Wls^fl/fl^* mice by flushing with αMEM medium (Welgene Inc.) using a 29-G needle-adhesive syringe at 4 weeks post-TBI. The cell suspension was filtered using a 70-μm cell strainer (BD Falcon, BD Biosciences), and the filtered cells were cultured in αMEM supplemented with 2 mM glutamine, antibiotics (100 IU/ml penicillin G and 100 μg/ml streptomycin), and 20% fetal bovine serum (HyClone Laboratories, Logan, UT, USA). On the second day, non-adherent cells were removed, and the remaining adherent cells were used as BM stromal cells (BMSCs, also known as BM-derived mesenchymal stem/stromal cells) [4,28]. BMSCs were cultured in the same medium, and after 12 days of additional incubation, the adherent cells were fixed with 10% formalin for 10 min and stained with 0.5% crystal violet dissolved in 100% methanol. BMSC-derived colonies containing more than 50 cells per colony were counted using an optical microscope.

### RNA isolation and qRT-PCR analysis

Total RNA was extracted from spleen-conserved cells using Trizol reagent (Invitrogen), and an RNA sample (1 μg) was used for cDNA synthesis with an AmpiGene™ cDNA synthesis kit (CAS:50-201-3813; Enzo Life Sciences, Inc., Farmingdale, NY, USA). qRT-PCR was done using Power SYBR^®^ Green PCR Master Mix (Applied Biosystems, Foster City, CA, USA) in an ABI StepOnePlus sequence detection system (Applied Biosystems). The thermocycling conditions were as follows: pre-denaturation at 95°C for 10 min and amplification using three-step cycles of denaturation at 95°C for 15 s, annealing at 60°C for 30 s, and extension at 72°C for 30 s, for 40 cycles. The oligonucleotide primers specific to *Wls* (forward-ACCGTGATGATAT GTTTTCTG and reverse-TACCACACCATAATGATG AA, NM_001356350.1), Wnt3a (forward-CACCACC GTCAGCAACAGCC and reverse-AGGAGCGTGTC ACTGCGAAAG, NM_009522.2), and Wnt5a (forward-CTCCTTCGCCCAGGTTGTTATAG and reverse-TGT CTTCGCACCTTCTCCAATG, NM_009524.4) were designed using Primer Express 3.0 (Applied Biosystems) to amplify products less than 200 bp in length. Glyceraldehyde 3-phosphate dehydrogenase (forward-GACGGCCGCATCTTCTTG and reverse-CACACCGA CCTTCACCAT, XM_017321385.1) was used as an internal marker during the quantification.

### ELISA

The protein levels of Wnt3a, Wnt5a, and DKK1 in BM and spleen lysates isolated from TBI-exposed *Wls^fl/fl^* and *Col-Cre*;*Wls^fl/fl^* mice were quantified using anti-mouse Wnt3a (OKEH03470), Wnt5a (OKEH04420), and DKK1 (OKEH04073) ELISA kits (Aviva Systems Biology, San Diego, CA, USA), respectively, at various times (0-72 h) after TBI exposure according to the manufacturer’s instructions.

### μCT analysis

*Wls^fl/fl^* and *Col-Cre*;*Wls^fl/fl^* mice were exposed to sub-lethal TBI at 4 weeks of age, and their long bones, including the cortical and trabecular bones, were scanned 4 weeks post-TBI using a desktop scanner (1076 Skyscan Micro-CT, Skyscan, Kontich, Belgium). The conditions were set at the maximum voltage of 100 kV and a 100-μA current with a 1-mm filter at 360° tomographic rotation (0.6° rotation step). Images were obtained at 18 μpixels, and data were analyzed using the SkyScan NRecon reconstruction package (Data Viewer, Bruker-μCT-Analyzer version 1.13, and CT Vol). A global thresholding algorithm was used as a uniform threshold. To analyze cancellous/trabecular bones, the regions of interest were positioned 0.25 mm proximity to the growth plate of the distal metaphysis, followed by selection of the region covering 2.5 mm proximally. Diaphysis cortical bones were evaluated by extending 0.5 mm proximally and distally from the midpoint of the femoral ends. Based on the constructed 3D images, we evaluated the values of bone-specific parameters in the trabecular zone: bone volume (BV, mm^3^), bone volume/tissue volume (BV/TV, %), trabecular thickness (Tb.Th., mm), trabecular number (Tb.N., 1/mm), porosity (Po, %), and bone mineral density (BMD, g/cm^3^).

### Measurement of blood cells

*Wls^fl/fl^* and *Col-Cre*;*Wls^fl/fl^* mice were exposed to sub-lethal TBI, and peripheral blood samples were isolated from mutants and littermate controls and collected into Vacutainer plastic tubes coated with K_2_EDTA at 12, 24, and 72 h post-TBI. We determined the levels of circulating white blood cells (WBC), lymphocytes, granulocytes, RBC, and platelets in the blood samples using an automated blood cell counter (Sysmex XE-2100; TOA Medical Electronics Co., Kobe, Japan).

### IHC

BM samples were collected from *Wls^fl/fl^* and *Col-Cre*;*Wls^fl/fl^* mice at various times (0-72 h or 5 day) of sub-lethal TBI, fixed overnight in 4% paraformaldehyde at 4°C, and decalcified in 17% EDTA. The tissue samples were dehydrated through a graded series of ethanol, embedded in paraffin, and sectioned at a thickness of 5 μm. Sectioned samples were incubated with anti-Wnt3a (ab28472; Abcam, Cambridge, UK), anti-Wnt5a (ab229200), anti-DKK1 (ab61034), or anti-fibroblast growth factor-21 (FGF21) primary antibody (#MA5-32652, Invitrogen), followed by incubation with secondary biotinylated antibodies (ab6788; Abcam). The sections were stained with a DAB Peroxidase Substrate Kit (CAS:SK-4100; Vector Laboratories, Burlingame, CA, USA) according to the manufacturer’s protocol and counterstained with Mayer’s hematoxylin. The sections were mounted and photographed using a microscope linked to a camera and image processing software (Leica Application Suite V4, Informer Technologies, Inc., Los Angeles, CA, USA). The cell area (%) that was positively stained with each of antibodies was evaluated using ImageJ software (NIH Clinical Center, Bethesda, MD, USA). Alternatively, the FGF21-specific DAB intensity was also determined by converting the quantified image intensity to optical density using the same software.

### Assay for BMSC proliferation

BMSCs were isolated from the BM of TBI-exposed and -unexposed *Wls^fl/fl^* and *Col-Cre*;*Wls^fl/fl^* mice. These cells were seeded onto a 96-multiwell culture plate (2 × 10^3^ cells/well) in growth medium and after various times (0-7 days) of incubation, the proliferation rate of the BMSCs was assessed using a Cell Counting Kit-8 (CAS:CK04; Dojindo Lab, Rockville, MD, USA) according to the manufacturer’s protocol.

### Osteogenic differentiation assay

To determine the effects of osteoblastic *Wls* deletion on the osteogenic differentiation of BMSCs under IR stress, BMSCs were isolated from sub-lethal TBI-exposed or -unexposed mutant and *Wls^fl/fl^* mice 4 weeks post-TBI and incubated in 24-well culture plates (10^5^ cells/well) in the presence and absence of 100 nM dexamethasone, 50 μM ascorbic acid, and 10 mM β-glycerophosphate (DAG). On day 21, the BMSCs were washed with phosphate buffered saline, fixed with 70% ethanol, and stained with 2% Alizarin red S (pH 4.2). The stained cells were treated with 10% cetylpyridinium chloride dissolved in 10 mM sodium phosphate (pH 7.0), and the absorbance of the dye was measured at 405 nm using an Emax precision microplate reader (Molecular Devices, San Jose, CA, USA).

### Statistical analyses

All data are expressed as the mean ± standard deviation and were analyzed using SPSS (ver. 16.0). In relation to the numbers of samples, unpaired Student’s *t*-test (n ≥ 6) or a non-parametric test (Wilcoxon *t*-test, n < 6) was used to determine significant differences between two sets of data. One-way ANOVA followed by Scheffe’s multiple range test was used for multiple comparisons among more than two groups. The Kolmogorov-Smirnov test was used to test the normality of data sets. A value of *p* < .05 was considered statistically significant.

## RESULTS

### Osteoblastic Wls ablation mediates the resistance of BM HSCs to TBI-induced oxidative stress and senescence

**Figure 1.**
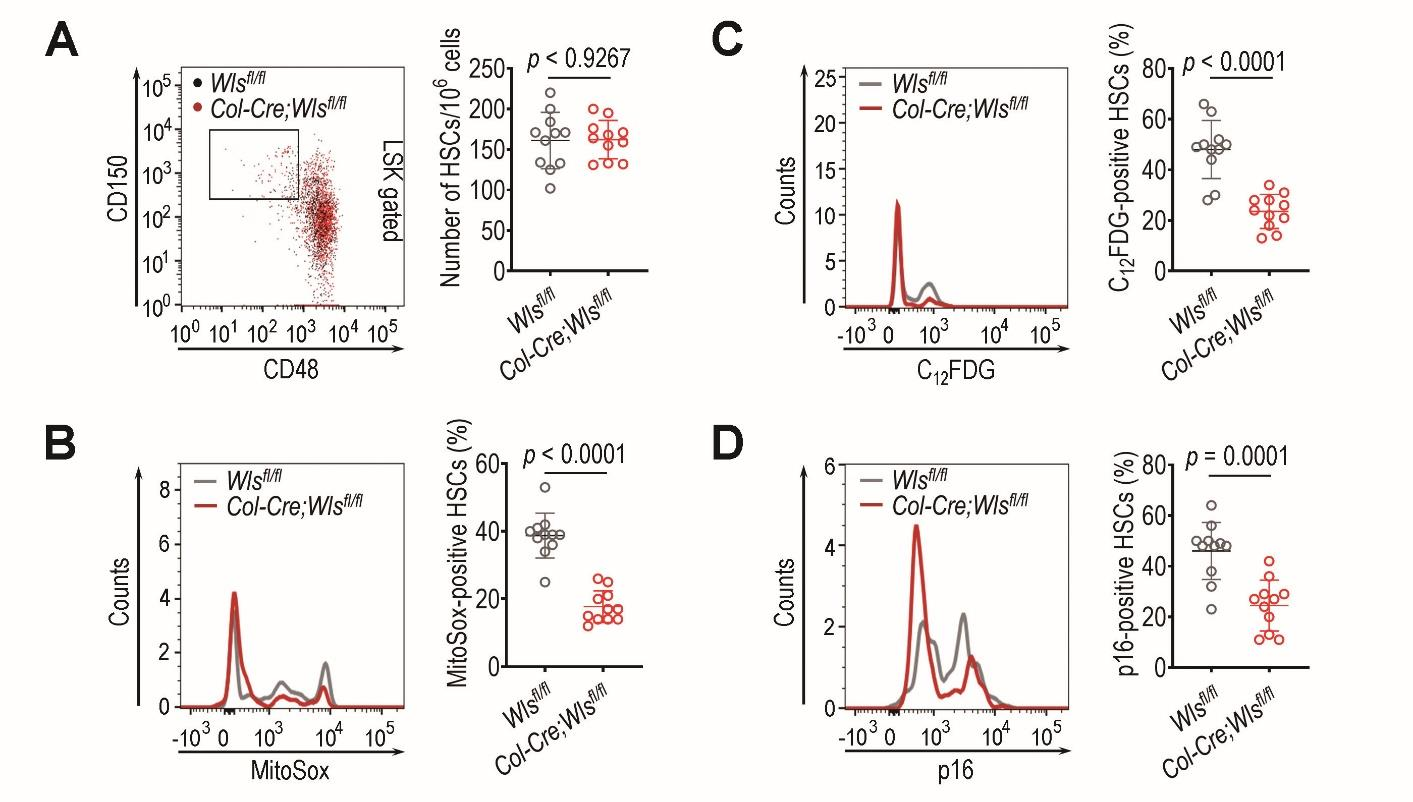
Deletion of *Wls* in *Col1a1*-expressing cells protects BM HSCs from TBI-mediated oxidative stress and senescence. Four-week-old *Wls^fl/fl^* and *Col-Cre;Wls^fl/fl^* mice were exposed to sub-lethal TBI (5 Gy), and (A) the BM frequency of HSCs and their numbers positive for (B) MitoSox, (C) C_12_FDG, or (D) p16^INK4a^ were evaluated by flow cytometry 4 weeks post-TBI (n = 11). The *p* values were determined by unpaired Student’s *t*-test.

Wnt signaling modulates the quiescence and self-renewal of HSCs in BM niches [28,29], where hematopoietic cells are prone to oxidative stress-associated senescence under IR stress [30]. We previously found that genetic *Wls* ablation in *Col1a1*-expressing cells did not induce HSC senescence or hematopoietic impairment at a young age [10]. Similarly, *Col-Cre*;*Wls^fl/fl^* mice showed a BM frequency and population (%) of HSCs positive for MitoSox, C_12_FDG, or p16^INK4a^ comparable to those of the littermate control (*Wls^fl/fl^*) mice at 4 weeks of age (Supplementary Fig. 1A-D). The frequency of BM HSCs in the *Col-Cre*;*Wls^fl/fl^* and *Wls^fl/fl^* mice was also not changed 4 weeks after sub-lethal TBI (5 Gy) (Fig. 1A). However, the *Wls^fl/fl^* mice exhibited significantly higher levels (%) of BM HSCs positive for MitoSox (*p* < 0.0001, Fig. 1B), C_12_FDG (*p* < 0.0001, Fig. 1C), and p16^INK4a^ (*p* = 0.001, Fig. 1D) than the *Col-Cre*;*Wls^fl/fl^* mice at the same time post-TBI. These results indicate that osteoblastic *Wls* ablation provides radioresistance to BM HSCs against TBI-mediated oxidative stress and senescence.

### Osteoblastic Wls ablation protects against TBI-induced abnormal hematopoietic development and maintains the potential of HPCs to form colonies

**Figure 2.**
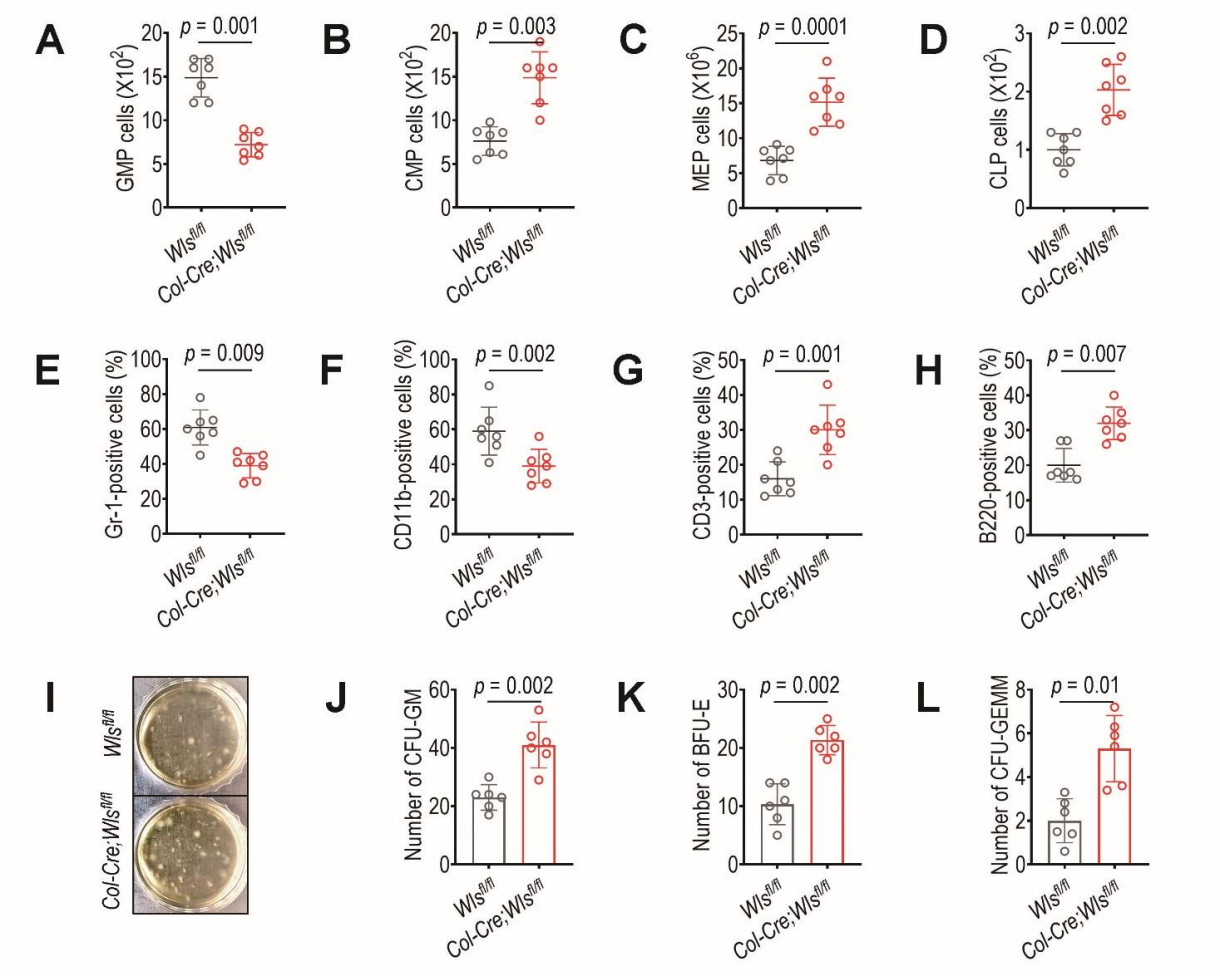
Ablation of *Wls* in *Col1a1*-expressing cells inhibits TBI-mediated skewing of BM HSCs toward myeloid progenitor and lineage and protects the colony forming potential of HPCs. Four-week-old *Wls^fl/fl^* and *Col-Cre;Wls^fl/fl^* mice were exposed to sub-lethal TBI, and at 4 weeks after TBI, the BM numbers of (A) GMP, (B) CMP, (C) MEP, and (D) CLP cells were determined by flow cytometry (n = 7). At the same time post-TBI, the proportions of (E) circulating granulocytes (positive to Gr-1), (F) monocytes (to CD11b), (G) T cells (to CD3), and (H) B cells (to B220) were flow cytometrically determined in PB (n = 7). BM HPCs were isolated from *Wls^fl/fl^* and *Col-Cre;Wls^fl/fl^* mice 4 weeks post-TBI and cultured in methylcellulose-based medium. After 12 days of incubation, the numbers of (I,J) CFU-GM, (K) BFU-E, and (L) CFU-GEMM were counted (n = 6). Photograph in panel I show a representative result from six different samples. The *p* values were determined by unpaired Student’s *t*-test.

On the basis that senescent HSCs preferentially differentiate into myeloid lineages instead of lymphoid lineages [31-33], we examined how genetic ablation of *Wls* in *Col1a1*-expressing cells influenced the proportions of myeloid progenitor and lineage cells after exposure to sub-lethal TBI. Similar to previous findings [10], in the absence of TBI, the *Wls^fl/fl^* mice exhibited levels of HPCs (Supplementary Fig. 2A-D), myeloid (Supplementary Fig. 2E and F), and lymphoid lineage cells (Supplementary Fig. 2G and H) and numbers of CFU-GM, BFU-E, and CFU-GEMM (Supplementary Fig. 2I-K) that were similar to those of *Col-Cre*;*Wls^fl/fl^* mice at 4 weeks of age. However, these comparable levels of hematopoietic cells between *Wls^fl/fl^* mice and *Col-Cre*;*Wls^fl/fl^* mice were tremendously affected in relation to the ablation of *Wls* in *Col2.3*-exprssin cells. Compared with non-TBI-exposed *Wls^fl/fl^* mice and *Col-Cre*;*Wls^fl/fl^* mice, relatively less GMP cells were found in the BM of *Col-Cre*;*Wls^fl/fl^* mice 4 weeks post-TBI (*p* = 0.001, Fig. 2A). The TBI-exposed *Wls^fl/fl^* mice showed significantly lower numbers of BM CMP (*p* = 0.003, Fig. 2B), MEP (*p* = 0.0001, Fig. 2C), and CLP (*p* = 0.002, Fig. 2D) cells than the TBI-exposed *Col-Cre*;*Wls^fl/fl^* mice. When the PB-derived lineage cells were counted 4 weeks after TBI, the *Col-Cre*;*Wls^fl/fl^* mice displayed cell numbers similar to those of non-TBI-exposed mutant mice (Supplementary Fig. 2E-H), but the numbers of cells positive for Gr-1 (*p* = 0.009, Fig. 2E), CD11b (*p* = 0.002, Fig. 2F), CD3 (*p* = 0.001, Fig. 2G), and B220 (*p* = 0.007, Fig. 2H) in the *Wls^fl/fl^* mice differed significantly from those in the *Col-Cre*;*Wls^fl/fl^* mice after TBI. TBI-exposed *Wls^fl/fl^* mice also had significantly lower activity in forming CFU-GM (*p* = 0.002, Fig. 2I and J), BFU-E (*p* = 0.002, Fig. 2K), and CFU-GEMM (*p* = 0.01, Fig. 2L) than the TBI-exposed mutant mice. These results indicate that osteoblastic *Wls* deletion itself does not impair hematopoietic development at a young age, but it protects hematopoietic cells from TBI-mediated disorders in hematopoietic differentiation and colony formation.

### Osteoblastic Wls ablation maintains donor cell repopulation capacity and protects recipient mice against lethal TBI

**Figure 3.**
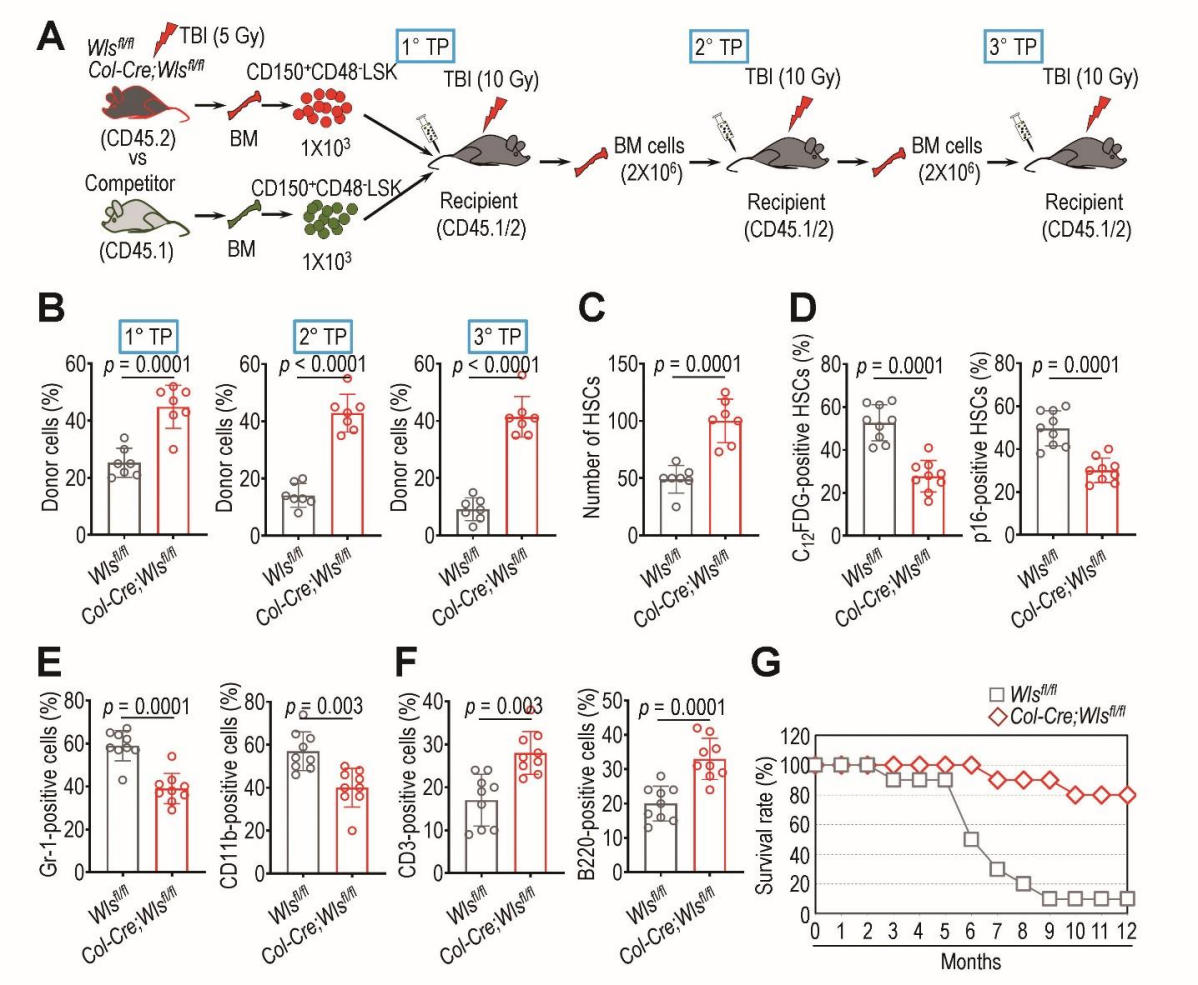
Ablation of *Wls* in *Col1a1*-expressing cells improves donor cell repopulation, BM engraftment, and lineage distribution of HSCs in lethally irradiated transplant recipients and protects recipient survival. (A) Scheme illustrating competitive and serial transplantation of BM HSCs or BM cells into recipients exposed to lethal TBI (10 Gy). (B) Long-term competitive repopulating activity of donor cells in the recipients of serial transplantation was determined by flow cytometry (n = 7). (C) Donor cell engraftment was assessed by measuring the number of BM HSCs in the 1° TP recipients 5 months post-transplantation using a flow cytometric analysis (n = 7). (D) Levels of C_12_FDG activity and p16^INK4a^ expression in donor-derived HSCs in the 1° TP recipients were measured after the removal of lineage cells by magnetic cell sorting (n = 9). (E and F) The proportions of circulating, donor cell-derived cells of the myeloid and lymphoid lineages were evaluated in PB from the 1° TP recipients 3 months post-transplantation (n = 9). (G) Mice exposed to lethal TBI received HSC transplants isolated from the BM of the 1° TP recipients, and the survival rate was monitored for up to 12 months post-transplantation (n = 10). The *p* values were determined by unpaired Student’s *t*-test.

We determined the effects of osteoblastic *Wls* ablation on donor cell repopulation in recipients by performing an *in vivo* competitive transplantation assay. To this end, we transplanted 10^3^ CD150^+^CD48^-^LSK cells derived from *Col-Cre*;*Wls^fl/fl^* or *Wls^fl/fl^* mice and an equal number of competitor mice-derived BM cells into lethally irradiated recipient mice (Fig. 3A). The BM cells (2 × 10^6^ cells) of those recipient mice were then serially transplanted into other conditioned recipients. HSCs derived from *Col-Cre*;*Wls^fl/fl^* mice showed greater donor cell repopulating ability (*p* ≤ 0.0001) than those from *Wls^fl/fl^* mice across all serial transplantations (Fig. 3B). These enhanced repopulating activities appeared to be related to the engrafted number of HSCs because the number of HSCs from the TBI-exposed mutant mice was significantly higher (*p* = 0.0001) in the BM of the primary-transplant recipients than in the corresponding controls (Fig. 3C). TBI-exposed mutant HSCs engrafted into the primary recipients displayed significantly lower levels (*p* = 0.0001) of SA-β-gal activity and p16^INK4a^ expression than TBI-exposed *Wls^fl/fl^* HSCs (Fig. 3D). Considering the skewing into myeloid differentiation of hematopoietic cells after TBI [10], the primary recipients of transplants from TBI-exposed mutant-derived HSCs revealed a relatively balanced distribution around 30-40% of PB-present myeloid (Fig. 3E) and lymphoid lineage cells (Fig. 3F), compared with the recipients of HSCs from the littermate controls. To further confirm the preservation of mutant HSC function under IR stress, we evaluated the survival rate of the conditioned recipients that were re-transplanted with donor-derived HSCs sorted from the primary recipients (Supplementary Fig. 3). Whereas the recipients of BM HSCs transplanted from TBI-exposed mutant mice exhibited an 80% survival rate 12 months post-transplantation, the recipients of HSCs derived from TBI-exposed *Wls^fl/fl^* mice exhibited only a 10% survival rate 9 months post-transplantation (Fig. 3G). These results support that osteoblastic *Wls* ablation confers radioresistance to BM HSCs because the recipients of mutant-derived HSCs or BM cells exhibited greater repopulating activity, engrafted HSCs, and survival rate, along with relatively lower senescence induction, than the recipients of cells derived from TBI-exposed *Wls^fl/fl^* mice.

**Figure 4.**
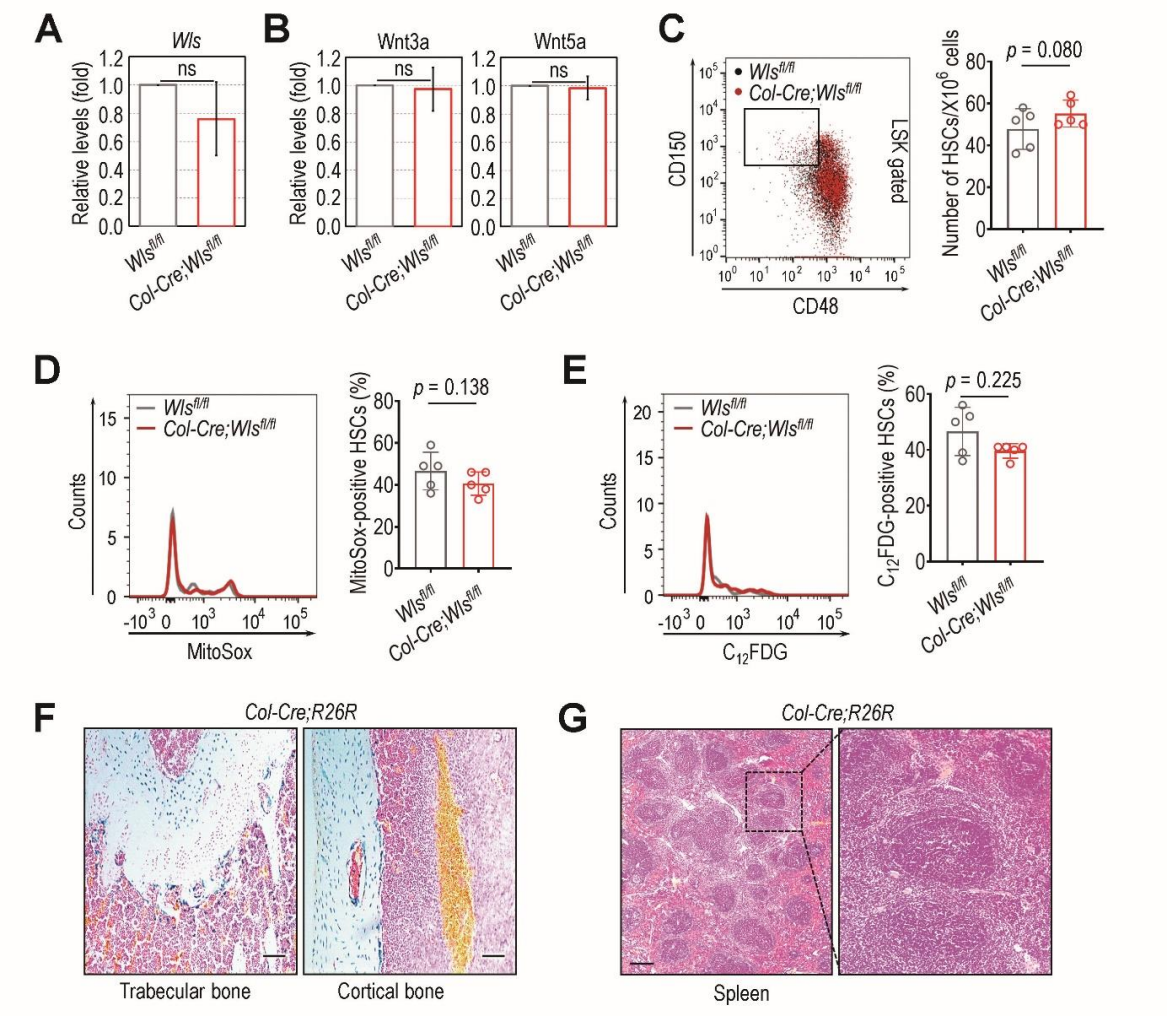
Deletion of *Wls* from *Col1a1*-expressing cells does not affect *Wls* expression or the secretion and senescence of HSCs in the spleens of TBI-exposed mice. Four-week-old *Wls^fl/fl^* and *Col-Cre;Wls^fl/fl^* mice were exposed to sub-lethal TBI, and (A) splenic *Wls* mRNA and (B) Wnt3a and Wnt5a protein levels in spleen supernatants were determined 4 weeks post-TBI by qRT-PCR and ELISA, respectively (n = 6). At the same time post-TBI, (C) the number of HSCs and (D) MitoSox- or (E) C_12_FDG-positive HSCs in the spleens of those mice were determined by flow cytometry (n = 5). *Col1a1-Cre* activity was determined in (F) the trabecular and cortical zones of the femur and (G) the spleens of mutant and control mice 4 weeks post-TBI. The representative images in panel (F) show regions stained with X-gal (blue), which indicate the active *Cre* recombinase sites in the sections. Here, the images exhibiting the X-gal-specific intensity at average level among five different samples were represented. The *p* values in panels A and B were determined by unpaired Student’s *t*-test. The *p* values in panels C-E were calculated using unpaired non-parametric Wilcoxon *t*-test. ns, not significant.

### Levels of splenic Wls, Wnt ligands, HSCs, and senescent HSCs are not directly affected by osteoblastic Wls ablation following TBI

We evaluated how osteoblastic *Wls* deletion affects *Wls* expression, the levels of Wnt3a and Wnt5a proteins, and several HSC phenotypes present in the spleen, an extramedullary hematopoietic organ, in TBI-exposed mutant and control mice. At 4 weeks post-TBI, the levels of *Wls* (Fig. 4A) and Wnt3a and Wnt5a ligands (Fig. 4B) in the spleens of *Col-Cre*;*Wls^fl/fl^* mice were comparable to those of *Wls^fl/fl^* mice. The *Col-Cre*;*Wls^fl/fl^* mice also did not display any distinct differences in the numbers of splenic HSCs (Fig. 4C) or the proportion of them positive for MitoSox (Fig. 4D) or C_12_FDG (Fig. 4E), compared with those in *Wls^fl/fl^* mice at the same time post-TBI. Interestingly however, the number of spleen-derived HSCs that positively exhibited the oxidative stress and senescence markers tended to be higher in the spleen than in the BM of both the mutant and control mice, regardless of TBI exposure (Fig. 1B and C, Supplementary Fig. 1B and C). To explore why HSCs derived from the BM and spleen exhibited different levels of oxidative stress and senescence, we performed X-gal staining using *Col2.3-Cre* mice crossed with *R26R* mice expressing a *LacZ* gene. Whereas the region stained blue, which is parallel with *Col1a1-Cre* activity, was apparent in the trabecular and cortical zones of the mice (Fig. 4F), spleen tissue did not show such a X-gal-positive region (Fig. 4G). These findings indicate that TBI-mediated phenotypes are distinctively expressed in the presence and absence of *Col1a1*-expressing cells and that osteoblastic Wnt can serve as an extrinsic signal mediating HSC senescence following TBI.

**Figure 5.**
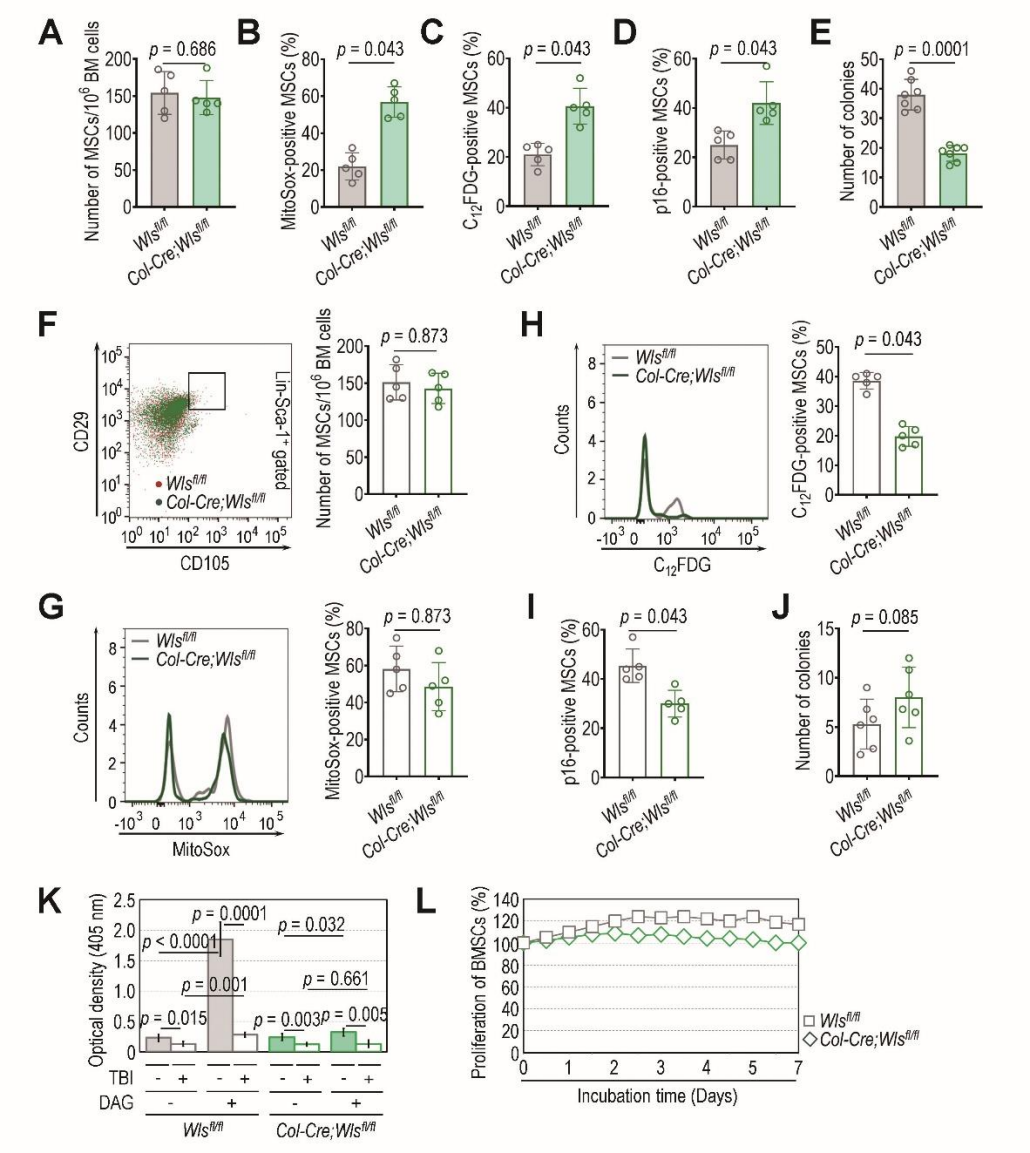
Deletion of *Wls* in *Col1a1*-expressing cells protects against TBI-mediated augmentation of senescent MSCs. (A) The frequency of MSCs in BM and the number positive for (B) MitoSox, (C) C_12_FDG, or (D) p16^INK4a^ in 4-week-old *Wls^fl/fl^* and *Col-Cre;Wls^fl/fl^* mice were analyzed by flow cytometry (n = 5). (E) The colony forming activity of BMSCs isolated from the mutants and littermate controls at 4 weeks of age was evaluated (n = 7). Four-week-old *Wls^fl/fl^* and *Col-Cre;Wls^fl/fl^* mice were exposed to sub-lethal TBI, and 4 weeks after TBI, (F) the frequency of BM MSCs, (G) MitoSox-, (H) C_12_FDG-, or (I) p16^INK4a^-positive MSCs, and (J) the colony forming activity of BMSCs were evaluated (n = 5 for F-I, n = 6 for J). (K) BMSCs isolated from TBI- or non-TBI-exposed *Wls^fl/fl^* and *Col-Cre;Wls^fl/fl^* mice were incubated in the presence and absence of DAG. After 21 days of incubation, the mineralization of these cells was evaluated by measuring the optical density at 405 nm (n = 7). (L) The proliferation rate of BMSCs isolated from TBI-exposed *Wls^fl/fl^* and *Col-Cre;Wls^fl/fl^* mice was monitored for 7 days by incubating them in growth medium. The *p* values in panels in panels A-D and F-I were calculated using unpaired non-parametric Wilcoxon *t*-test. The *p* values in panels E, J, and K were determined by unpaired Student’s *t*-test.

**Figure 6.**
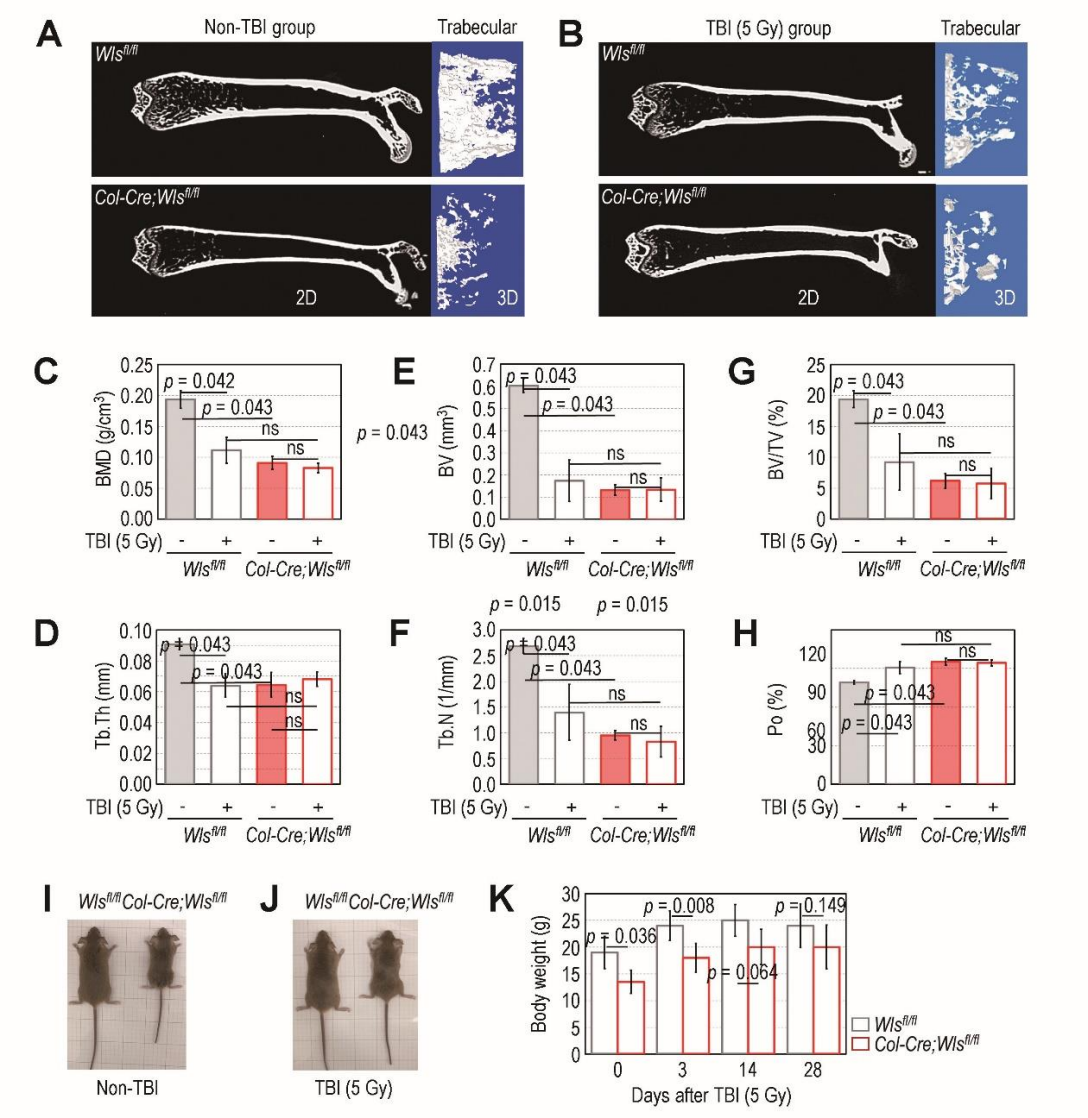
Deletion of *Wls* in *Col1a1*-expressing cells inhibits TBI-mediated enhancement of bone mass loss. Femoral bones (2D image) with magnified trabecular zones (3D image) in (A) non-TBI- and (B) TBI-exposed *Wls^fl/fl^* and *Col-Cre;Wls^fl/fl^* mice were analyzed by μCT when they were 8 weeks of age. For this experiment, the mouse groups for TBI were exposed to sub-lethal TBI when they were 4 weeks of age, and representative results exhibiting average BMD values among five different samples per group are shown. Values of (C) BMD (g/cm^3^), (D) Tb.Th. (mm), (E) BV (mm^3^), (F) Tb.N. (1/mm), (G) BV/TV (%), and (H) Po (%) were calculated based on the constructed 3D images (n = 5). Photographs showing (I) *Wls^fl/fl^* and *Col-Cre;Wls^fl/fl^* mice at 4 weeks of age and (J) after an additional 2 weeks of TBI. (K) The body weights (g) of the *Wls^fl/fl^* and *Col-Cre;Wls^fl/fl^* mice were monitored on the indicated days after TBI (n = 6). The *p* values in panels C-H were determined by non-parametric Wilcoxon *t*-test, whereas the values in panel K were by unpaired Student’s *t*-test. ns, not significant.

### TBI induces impaired function and senescence among MSCs but does not accelerate the cellular damage caused by osteoblastic Wls ablation

Similar to our previous findings [10], osteoblastic *Wls* deletion did not change the frequency of MSCs in BM (*p* = 0.686, Fig. 5A), but it did increase significantly the number of MSCs positive for MitoSox (*p* = 0.043, Fig. 5B), C_12_FDG (*p* = 0.043, Fig. 5C), and p16^INK4a^ (*p* = 0.043, Fig. 5D) and reduced the potential of BMSCs to form colonies (*p* = 0.0001, Fig. 5E). The frequency of MSCs in BM (Fig. 5F) and the level (%) of MitoSox-positive MSCs (Fig. 5G) in the *Col-Cre*;*Wls^fl/fl^* mice were comparable with those in the *Wls^fl/fl^* mice following TBI. However, significantly higher levels (%) of BM MSCs positive for C_12_FDG (*p* = 0.043, Fig. 5H) or p16^INK4a^ (*p* = 0.043, Fig. 5I) were found in TBI-exposed *Wls^fl/fl^* mice compared with the levels in TBI-exposed *Col-Cre*;*Wls^fl/fl^* mice. The colony forming potential of BMSCs derived from TBI-exposed *Col-Cre*;*Wls^fl/fl^* and *Wls^fl/fl^* mice did not differ much (Fig. 5J). DAG-mediated enhancement of *in vitro* mineralization was found in cultures of BMSCs derived from *Wls^fl/fl^* mice but not *Col-Cre*;*Wls^fl/fl^* mice, whereas that enhancement was not seen in cells isolated from TBI-exposed control and mutant mice (Fig. 5K). Furthermore, BMSCs derived from TBI-exposed *Wls^fl/fl^* mice did not show incubation time-relative proliferation compared with cells from the TBI-exposed mutant mice (Fig. 5L). Our results indicate that TBI causes oxidative stress and senescence in the BM MSCs of *Wls^fl/fl^* mice, and osteoblastic *Wls* ablation partially corrects TBI-mediated MSC senescence without having significant effects on the ROS accumulation, colony forming potential, osteogenic differentiation, or proliferation of MSCs or BMSCs. Thus, it is likely that, compared with MSCs, Wnt signaling more sensitively affects the fate and functions of BM HSCs following TBI.

**Figure 7.**
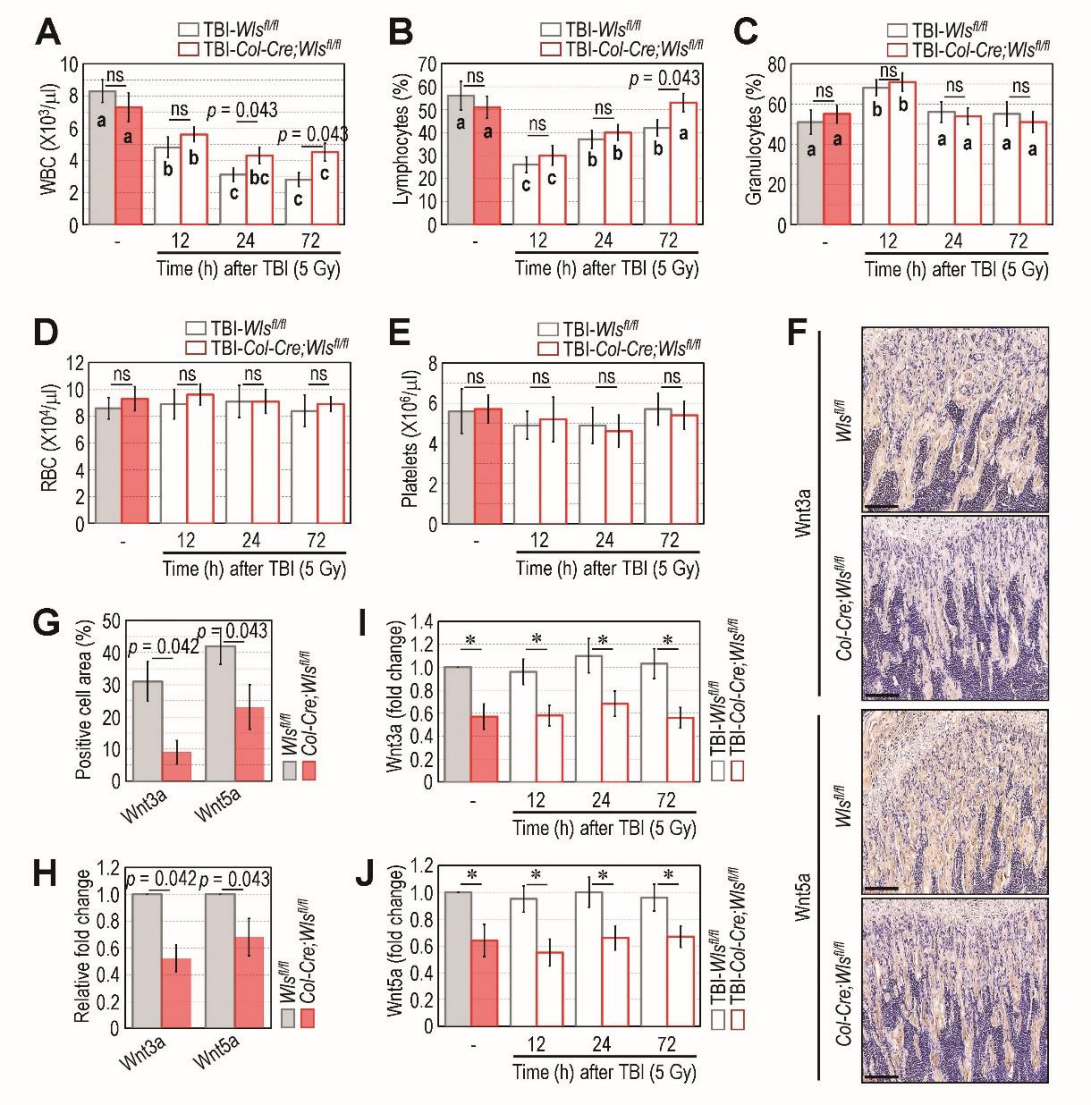
TBI acutely reduces the numbers of peripheral WBCs and lymphocytes but not the BM levels of Wnt ligands, and that is ameliorated by osteoblastic *Wls* depletion. The levels of circulating (A) WBCs, (B) lymphocytes, (C) granulocytes, (D) RBCs, and (E) platelets in the mouse groups were measured using an automated complete blood cell counter at the indicated times (h) after TBI (n = 5). (F) The BM levels of Wnt3a and Wnt5a in *Wls^fl/fl^* and *Col-Cre;Wls^fl/fl^* mice were evaluated by IHC when they were 4 weeks of age. Representative data from five different samples are shown. Scale bars = 100 μm. (G) The area (%) positively stained with Wnt3a or Wnt5a in the IHC assay was calculated (n = 5). (H) Levels of Wnt3a and Wnt5a mRNA in whole BM lysate were determined by qRT-PCR (n = 5). Protein levels of (I) Wnt3a and (J) Wnt5a in the whole BM lysate were determined by ELISA at the indicated times after TBI (n = 5). The *p* values in all panels were determined by non-parametric Wilcoxon *t*-test. The superscripts^a-c^ indicate significant differences among the groups compared with the value of non-TBI control or mutant group by ANOVA. The symbol ‘*’ in panels I and J indicate significant difference at *p* < 0.043 by the Wilcoxon *t*-test. ns, not significant.

### TBI severely impairs the BM microenvironment in Wls^fl/fl^ mice but does not enhance BM injury caused by osteoblastic Wls ablation

Compared with the *Wls^fl/fl^* mice, the *Col-Cre*;*Wls^fl/fl^* mice had decreased bone mass in the cortical and trabecular zones at 4 weeks of age (Fig. 6A). Bone mass accrual in the *Wls^fl/fl^* mice was evidently reduced following TBI, when the trabecular bone mass of the control mice tended to be similar to that in TBI-exposed *Col-Cre*;*Wls^fl/fl^* mice (Fig. 6B). The values of the bone parameters, BMD (gm/cm^3^) (Fig. 6C), Tb.Th. (mm) (Fig. 6D), BV (mm^3^) (Fig. 6E), Tb.N. (1/mm) (Fig. 6F), BV/TV (%) (Fig. 6G), and Po (%) (Fig. 6H), in the trabecular bone of TBI-exposed *Wls^fl/fl^* mice changed to levels similar to those of TBI-exposed *Col-Cre*;*Wls^fl/fl^* mice. In this experiment, we found no distinct differences in the values of bone parameters between TBI exposed- and -unexposed *Col-Cre*;*Wls^fl/fl^* mice (Fig. 6C-H). The *Col-Cre*;*Wls^fl/fl^* mice also exhibited a smaller body size than the *Wls^fl/fl^* mice at 4 weeks of age (Fig. 6I), but that difference gradually disappeared 2 weeks after TBI (Fig. 6J). No significant difference in body weight (g) between the control and mutant mice was found 2 (*p* = 0.064) and 4 weeks (*p* = 0.149) after TBI (Fig. 6K). These results indicate that TBI exposure diminishes bone mass accrual in *Wls^fl/fl^* mice, but osteoblastic *Wls* ablation protects mice from that TBI-mediated bone mass loss, contributing to a recovery of delayed body growth.

### TBI acutely impairs blood cell composition without altering BM levels of Wnt ligands and DKK1, whereas osteoblastic Wls ablation promotes the recovery of TBI-induced hematopoietic injury

TBI can acutely and severely disrupt BM maintenance of HSCs and the composition of circulating blood cells. To further explore the effects of sub-lethal TBI in regard to osteoblastic *Wls* ablation, we examined the frequency of BM HSCs and MSCs and measured several types of circulating blood cells in the mutant and control mice early (12, 24, and 72 h) after TBI. We also determined the BM levels of Wnt3a, Wnt5a, and DKK1 at those same times. The frequencies of BM HSCs and MSCs were not changed at the indicated times following TBI (Supplementary Fig. 4A and B). As expected, the circulating levels of WBCs (Fig. 7A), lymphocytes (Fig. 7B), and granulocytes (Fig. 7C), but not RBCs (Fig. 7D) or platelets (Fig. 7E), changed acutely following TBI regardless of *Wls* ablation. However, as shown in Fig. 7A and 7B, the TBI-mediated decrease in peripheral WBCs tended to be more severe in the *Wls^fl/fl^* mice than the *Col-Cre*;*Wls^fl/fl^* mice, and the peripheral recovery of lymphocytes in the TBI-exposed mutant mice was greater than that in the TBI-exposed control mice. The IHC (Fig. 7F and G) and ELISA (Fig. 7H) results support that the *Wls^fl/fl^* mice had higher expression of Wnt3a and Wnt5a in trabecular bone than the *Col-Cre*;*Wls^fl/fl^* mice. The expression patterns of these Wnt ligands were not significantly changed by TBI (Fig. 7I and J). Further, the IHC and ELISA data show no differences in the intensity or expression of DKK1 in BM (Supplementary Fig. 5A-C). Furthermore, the level of FGF21, a potential biomarker for mitochondrial disorders [34], in the BM of *Col-Cre*;*Wls^fl/fl^* mice was comparable with that in *Wls^fl/fl^* mice, and these levels were also not changed by TBI (Supplementary Fig. 6A and B). All of these results suggest that osteoblastic *Wls* deletion promotes the peripheral regeneration of lymphoid lineage cells after TBI, whereas the expression and secretion of Wnt ligands, DKK1, and FGF21 are not directly affected by TBI.

## DISCUSSION

BM provides stem cell niches in which Wnt signaling plays important roles in modulating the quiescence and self-renewal of HSCs [28,29]. It was previously assumed that osteoblastic Wnt is not an indispensable factor in the maintenance and self-renewal of BM HSCs at a young age [35]. However, our results show that exposure to TBI at 4 weeks of age distinctively modulated the fate and functions of BM HSCs in the presence and absence of *Wls* in *Col1a1*-expressing osteoblastic cells. Unlike the *Wls^fl/fl^* littermate controls, the *Col-Cre*;*Wls^fl/fl^* mutant mice exhibited hematopoietic radioprotection, with significantly lower levels of ROS accumulation, SA-β-gal activity, and p16^INK4a^ expression in BM-conserved HSCs, as well as higher levels of colony forming and long-term repopulating potential than the control mice. Transplantation of BM HSCs derived from the mutant mice improved the survival of lethally irradiated recipients compared with transplantation of cells from the control mice. Dissimilar to the BM and BM HSCs, spleen tissue revealed unchanged levels of Wnt ligands, along with higher levels of MitoSox- and C_12_FDG-positive HSCs than were present in the BM. These differences between the BM and spleen are considered to be due to the presence of *Col1a1*-expressing osteoblastic cells. Taken together, our results demonstrate that TBI sensitively and tremendously impairs the functions of BM HSCs by causing severe oxidative stress and senescence, and osteoblastic *Wls* deficiency provides hematopoietic radioprotection and regeneration.

Dissimilar to HSCs, osteoblastic *Wls* ablation itself severely impaired the fate and functions of BM-conserved MSCs and caused declines in bone mass accrual at 4 weeks of age [10]. Our current findings also support those functional defects in MSCs because the *Col-Cre*;*Wls^fl/fl^* mice had higher levels of oxidative stress and senescence markers than the *Wls^fl/fl^* mice. However, exposure to TBI increased the number of MSCs positive for senescence markers in *Wls^fl/fl^* mice more greatly than it did in the *Col-Cre*;*Wls^fl/fl^* mice. TBI exposure also reduced the bone mass accrual and bone parameter values in the *Wls^fl/fl^* mice but not in the *Col-Cre*;*Wls^fl/fl^* mice. The mutant mice did not exhibit any alterations in bone mass or microenvironmental parameters after TBI. Given that bone mass accrual was further severely diminished in the *Col-Cre*;*Wls^fl/fl^* mice in regard to age [10], bone mass in the TBI-exposed mutants at 4 weeks of age was still higher than that of the normally aging mutant mice. Thus, we consider that osteoblastic deficiency of Wnt ligands not only renders at least partial radioprotection to BM MSCs or osteoprogenitor cells, but also prevents excessive TBI-induced senescence of MSCs and bone mass loss.

Similar to the TBI-exposed *Wls^fl/fl^* mice, BMSCs derived from the TBI-exposed *Col-Cre*;*Wls^fl/fl^* did not show colony forming potential or DAG-enhanced mineralization. These results indicate that the properties of BMSCs exposed to TBI differ in regard to the experimental conditions, namely *in vivo*, *ex vivo*, and *in vitro*. A previous report showed that although an age-dependent increase in radioresistance occurred in the HPCs of irradiated mice, those same cells exposed to *in vitro* irradiation did not show that resistance [36]. It was reported that dissimilar to morphology and phenotype, TBI-exposed BMSCs or conditioned recipient-derived BMSCs exhibit lower potentials to proliferate and differentiate with a clonal cytogenetic abnormality compared with non-TBI BMSCs [37]. It is important to note that *Col2.3* is specific to almost differentiated or mature osteoblasts, and thus the ablation of *Wls* in the *Col2.3-Cre* mice causes osteoblastic niches- and osteoblast-specific absence of Wnt ligands rather than BM-derived undifferentiated stem cells such BMSCs and HPCs [23,24,38]. These indicate that not only TBI induces sensitively MSC injury and long-term BM complications, but also the inconsistent results of MSC damages between in vivo and ex vivo are due to the different phenotypes of BM-derived cells in secreting Wnt ligands. Further, the radioresistance or radiosensitivity of MSCs tended to be dependent on the BM microenvironment and other extrinsic molecules, rather than on the intrinsic characteristics of the MSCs themselves. It also suggests that osteoblastic Wnt is an important extrinsic factor that modulates the fate and functions of MSCs following TBI. Overall, considering TBI applied in HSC transplantation (HSCT) and HSCT-associated complications to stem cells, we consider that in vivo results reflect further exactly the actual roles of Wnt signaling on BM and MSCs in TBI-exposed patients.

DKK1, an inhibitor of Wnt signaling, is expressed and secreted by osteolineage cells under the control of the osteogenic transcriptional factor osterix [39]. It was reported that directly adding DKK1 into cultures of irradiated HSCs recovered their repopulating potential [21]. Systemic DKK1 supplementation also improved hematopoietic recovery and survival in TBI-exposed mice [21]. Those improvements are considered to be directly associated with DKK1-mediated suppression of ROS accumulation and senescence induction in HSCs. Those effects of DKK1 are consistent with the limitation of TBI-mediated senescence and functional impairment in BM HSCs conferred by osteoblastic *Wls* deletion. It is also suggested that during homeostasis, DKK1 promotes the expansion of a pool of myeloid progenitor cells [21]. We previously found that a long-term deficiency of osteoblastic Wnt skewed hematopoietic development toward myeloid progenitor and lineage cells [10]. However, our current findings reveal that osteoblastic *Wls* depletion itself did not alter the DKK1 level in BM, regardless of TBI exposure. This result indicates that the hematopoietic radioprotection offered by osteoblastic *Wls* depletion is not directly associated with DKK1-mediated signaling.

FGF21, one of the metabolism-related diagnostic cytokines, exerts a key role in glucose homeostasis and serves as a specific biomarker in mitochondrial dysfunctions [34]. A report showed that FGF21 regulates mitochondrial dynamics by activating AMPK signaling, and siFGF21 transfection induces ROS generation and senescence of BMSCs [40]. Overexpression of FGF21 in BMSCs enhanced their homing to injury site in animal model of traumatic brain injury thereby indicating a possible approach for MSC-based therapy in the injury [41]. Here we found that level of FGF21 in the BM was not changed by the ablation of *Wls* in *Col2.3*-expressing cells or in combination with sub-lethal TBI. This result is similar to our previous findings showing that cell cycle progression and number of Annexin V/propidium iodide-positive cells (%) in the *Col-Cre*;*Wls^fl/fl^* mice-derived BMSCs at 4 weeks of age were comparable with those in *Wls^fl/fl^*-derived cells [10]. Taken as a whole, this study demonstrates that unlikely to ROS accumulation and senescence induction, deletion of *Wls* in *Col2.3*-expressing cells or in combination with TBI does not directly dysregulate the mitochondrial and cellular stress marker, FGF21.

Various kinds of chemokines and cytokines have been associated with radioprotection or radiosensitization. The increased secretion of epidermal growth factor from BM endothelial cells is considered to be an indirect mechanism promoting DKK1-mediated hematopoietic regeneration [21]. Although the SDF-1/CXCR4 signaling axis is important for BM retention and the development of HSCs [42], deletion of the gene encoding SDF-1 in BM osteoblasts did not affect HSC content during homeostasis [43,44]. The levels of *Csf1*, *Csf2, Cxcl12, Fgf1, Fgf7, Il1a, Il1b*, and *Kitl* that have been reported to be radioprotective can be enhanced in BM tissue after TBI depending on age [38]. Cell cycle regulatory genes such as cyclin D1 are also upregulated in LSK cells derived from irradiated mice [45]. Those reports indicate that various endo- and exogeneous factors could be associated with hematopoietic radioprotection. Further experiments are needed to clarify which chemokines or cytokines secreted in the BM or osteoprogenitor cells are directly or indirectly associated with radioprotection in TBI-exposed *Col-Cre*;*Wls^fl/fl^* mice.

**Figure 8.**
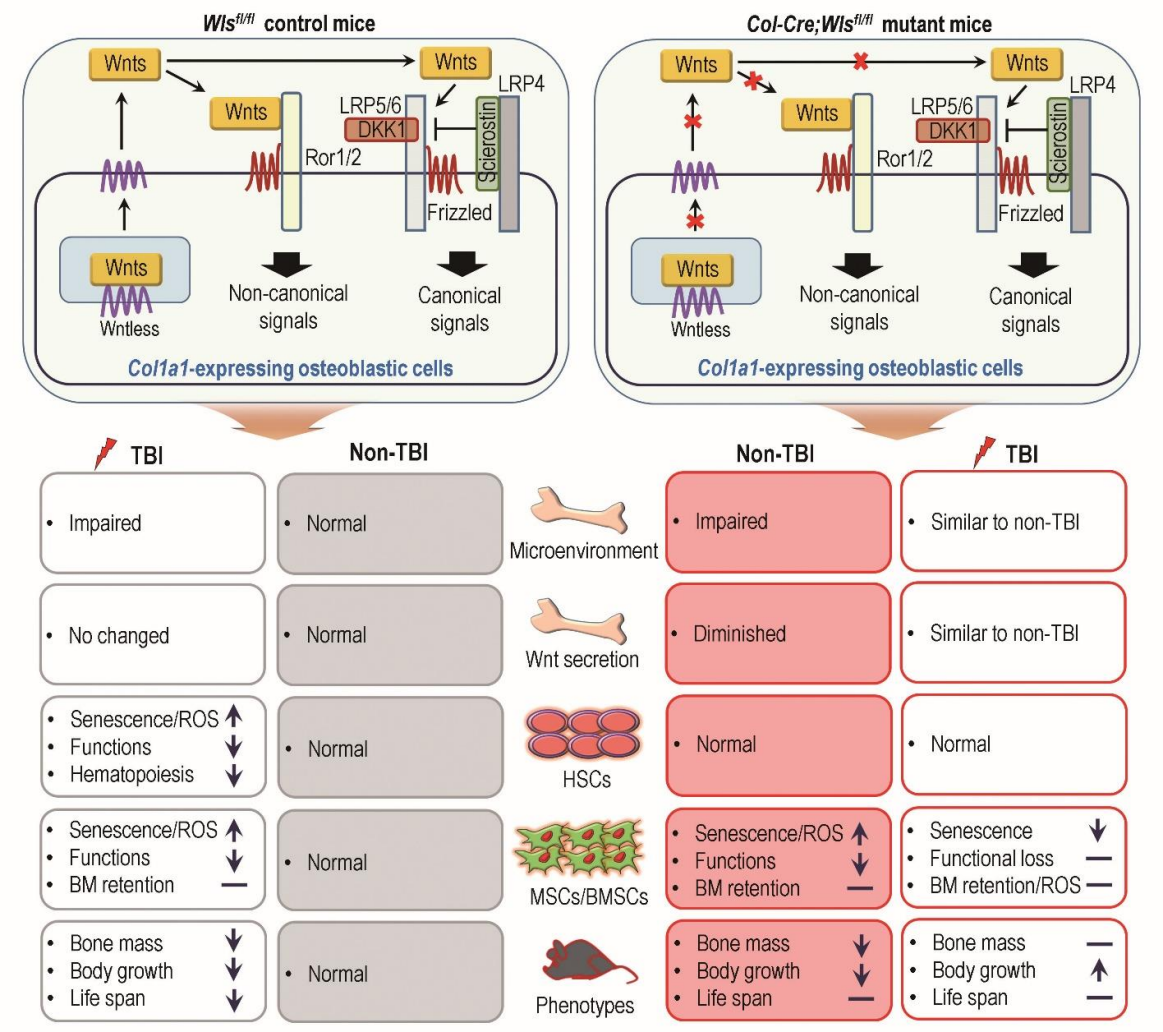
Schematic illustration of the roles played by osteoblastic *Wls* ablation in TBI-mediated alterations in the BM microenvironment, bone mass accrual, fate and functions of BM HSCs and MSCs, and survival.

On the other hand, several studies have shown controversial results about the effect of β-catenin on BM HSCs [46-48]. It is suggested that non-canonical Wnt signaling maintains quiescent HSCs via inhibition of canonical Wnt signaling [49]. A recent report highlights that the suppression of canonical Wnt signaling accelerated the *ex vivo* maintenance and proliferation of HSCs and increased HPC numbers in zebrafish [50]. Although the role of canonical Wnt in the fate and function of BM HSCs remains unclear, these reports together with our findings strongly indicate that deficiency of canonical Wnt ligands and decreased activation of canonical Wnt signaling are required for hematopoietic radioprotection. Osteoblastic *Wls* depletion might non-specifically diminish the secretion of Wnt ligands involved in canonical or non-canonical Wnt signaling. Additional experiments are needed to verify whether canonical, non-canonical, or both types of Wnt signaling are essential for radioprotection of the hematopoietic system and BM microenvironment in regard to osteoblastic *Wls* ablation.

In summary, our findings support that osteoblastic *Wls* ablation renders BM HSCs and MSCs resistant to TBI-mediated senescence and functional damage and also improves the BM microenvironment and survival under IR stress (Fig. 8). As TBI is often used in HSCT and contributes to various cellular and tissue injuries, our current findings along with previous reports [20,21-23] indicate that blocking osteoblast-specific Wnt signaling or Wnt ligand secretion in *Col1a1*-expressing osteoblasts is to be an attractive approach in inhibiting TBI-mediated long-term complications and abnormal development of stem cells. It will be worthy of further study to determine whether osteoblastic Wnt, especially that secreted by *Col1a1*-expressing cells, differently modulate BM HSCs and MSCs following TBI. It will also be intriguing to verify whether modulating Wnt signaling in the BM can serve as a supplemental remedy for patients who suffer from long-term residual BM injury with HSC senescence following TBI.

## Supplementary Materials

The Supplementary data can be found online at: www.aginganddisease.org/EN/10.14336/AD.2021.1026.
