# Supplementary material for: Osteoblastic Wls Ablation Protects Mice from Total Body Irradiation-Induced Impairments in Hematopoiesis and Bone Marrow Microenvironment
Source: Aging Dis. 2023 Jun 1;14(3):919–36. doi: 10.14336/AD.2022.1026 (PMC10187694; doi:10.14336/AD.2022.1026)
Supplement: Supplementary file 1 — The Supplementary data can be found online at: www.aginganddisease.org/EN/10.14336/AD.2021.1026. [file AD-14-3-919-s.pdf]

SUPPLEMENTARY DATA

Osteoblastic *Wls* Ablation Protects Mice from Total Body Irradiation–Induced Impairments in Hematopoiesis and Bone Marrow Microenvironment

Hyun-Jaung Sim[1,2#], Han-Sol So[1#], Sher Bahadur Poudel[3#], Govinda Bhattarai[2,4], Eui-Sic Cho[2,4], Jeong-Chae Lee[1,4*], Sung-Ho Kook[1,2*]

© 2022. Sim HJ et al. Published online at http://www.aginganddisease.org/EN/10.14336/AD.2022.1026

SUPPLEMENTARY DATA

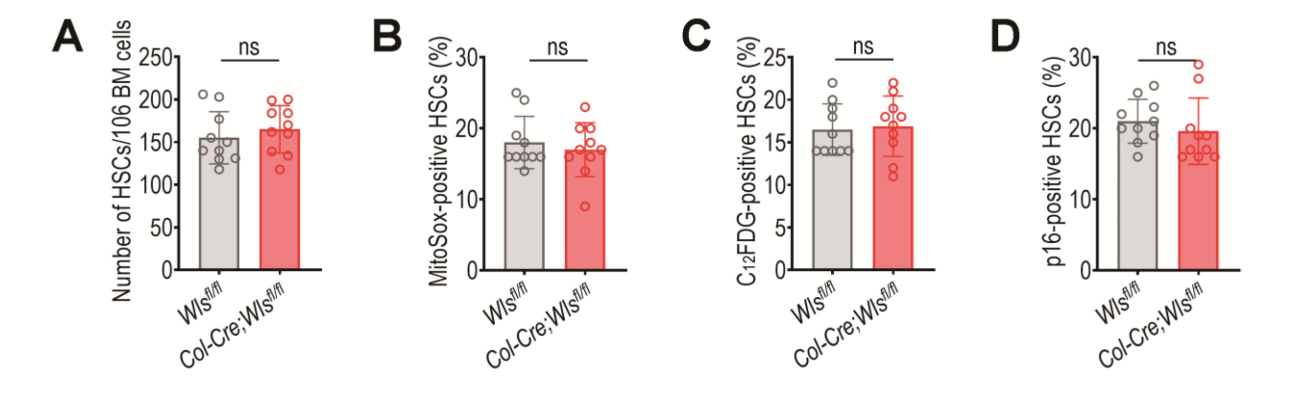

Supplementary Figure 1. Osteoblastic *Wls* deletion does not alter BM number of HSCs and expression levels of oxidative stress- or senescence-specific markers in these cells at 4 weeks of age. **(A)** BM frequency of HSCs and numbers of HSCs positive to **(B)** MitoSox, **(C)** C_{12}FDG, or **(D)** p16^{INK4a} in *Wls*$^{fl/fl}$ and *Col2.3-Cre;Wls*$^{fl/fl}$ mice at 4 weeks of age were analyzed by flow cytometry (n = 10). The significant differences were determined by unpaired Student *t*-test. ns, not significant.

© 2022. Sim HJ et al. Published online at http://www.aginganddisease.org/EN/10.14336/AD.2022.1026

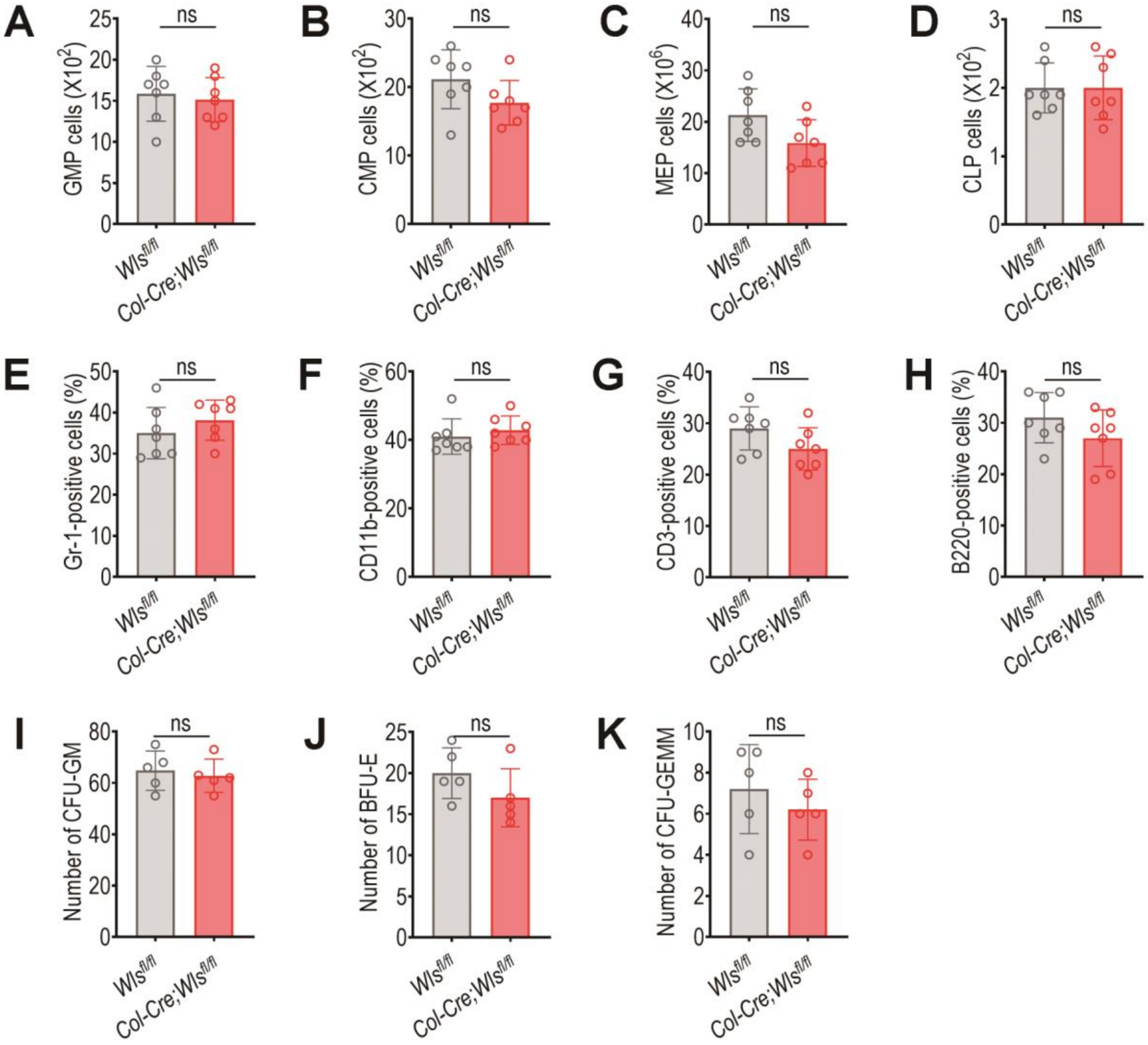

Supplementary Figure 2. Osteoblastic *Wls* ablation does not change hematopoietic development and colony forming potentials of HPCs at young stage. Numbers of **(A)** GMP, **(B)** CMP, **(C)** MEP, and **(D)** CLP cells in *Wls$^{fl/fl}$* and *Col2.3-Cre;Wls$^{fl/fl}$* mice at 4 weeks of age were determined by flow cytometry (n = 7). At the same age, proportions of peripheral cells positive to **(E)** Gr-1, **(F)** CD11b, **(G)** CD3, or **(H)** B220 were determined (n = 7). In addition, BM HPCs isolated from *Wls$^{fl/fl}$* and *Col2.3-Cre;Wls$^{fl/fl}$* mice at 4 weeks of age were cultured in methylcellulose-based medium. After 12 days of incubation, numbers of **(I)** CFU-GM, **(J)** BFU-E, and **(K)** CFU-GEMM were counted (n = 5). The significant differences in panels A–H were determined by unpaired Student *t*-test, whereas the values in panels I-K were by non-parametric Wilcoxon *t*-test. ns, not significant.

© 2022. Sim HJ et al. Published online at http://www.aginganddisease.org/EN/10.14336/AD.2022.1026

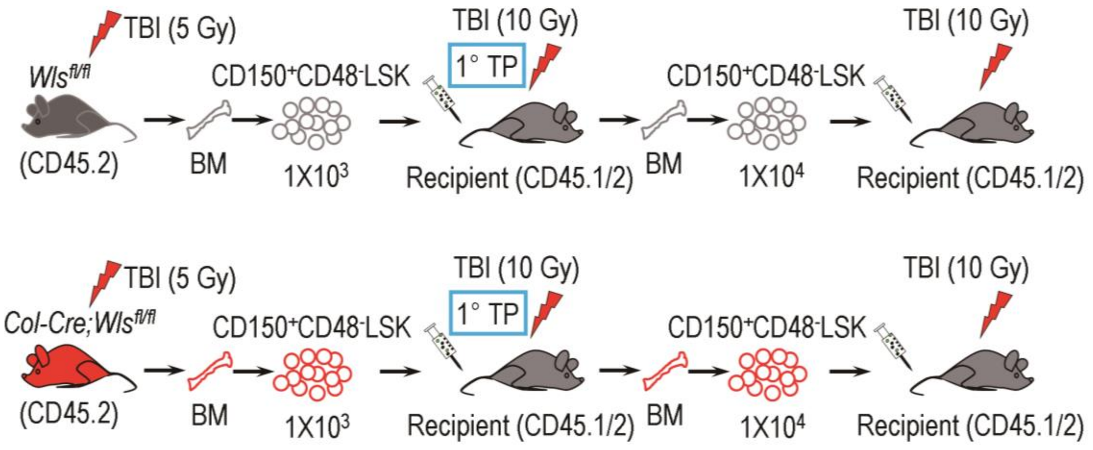

Supplementary Figure 3. A schematic illustration exhibiting serial transplantation of BM HSCs into lethally irradiated recipients for survival assay.

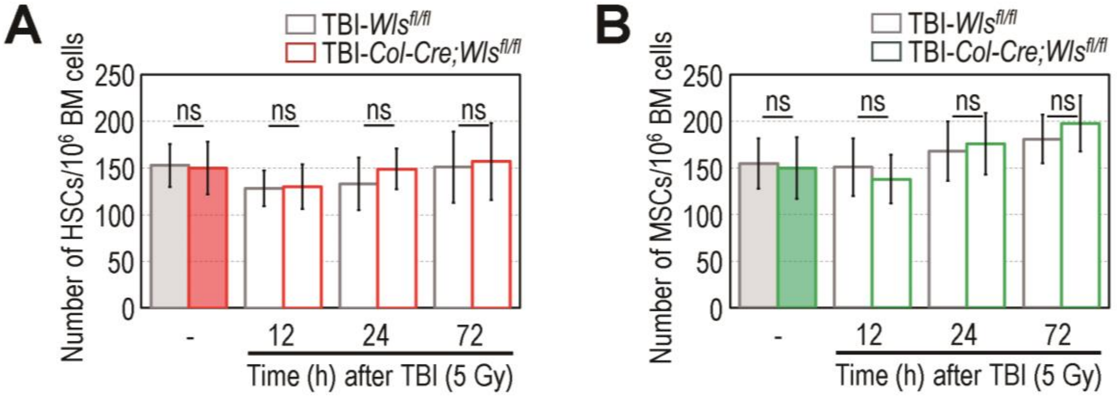

Supplementary Figure 4. TBI does not induce any acute changes in numbers of BM HSCs and MSCs regardless of the presence and absence of osteoblastic *Wls*. *Wls*$^{fl/fl}$ and *Col2.3-Cre;Wls*$^{fl/fl}$ mice at 4 weeks of age were exposed to sub-lethal TBI. The frequencies of **(A)** BM HSCs and **(B)** MSCs in these mice were determined by flow cytometry at the indicated times following TBI (n = 5). The significant differences were determined by non-parametric Wilcoxon *t*-test. ns, not significant.

© 2022. Sim HJ et al. Published online at http://www.aginganddisease.org/EN/10.14336/AD.2022.1026

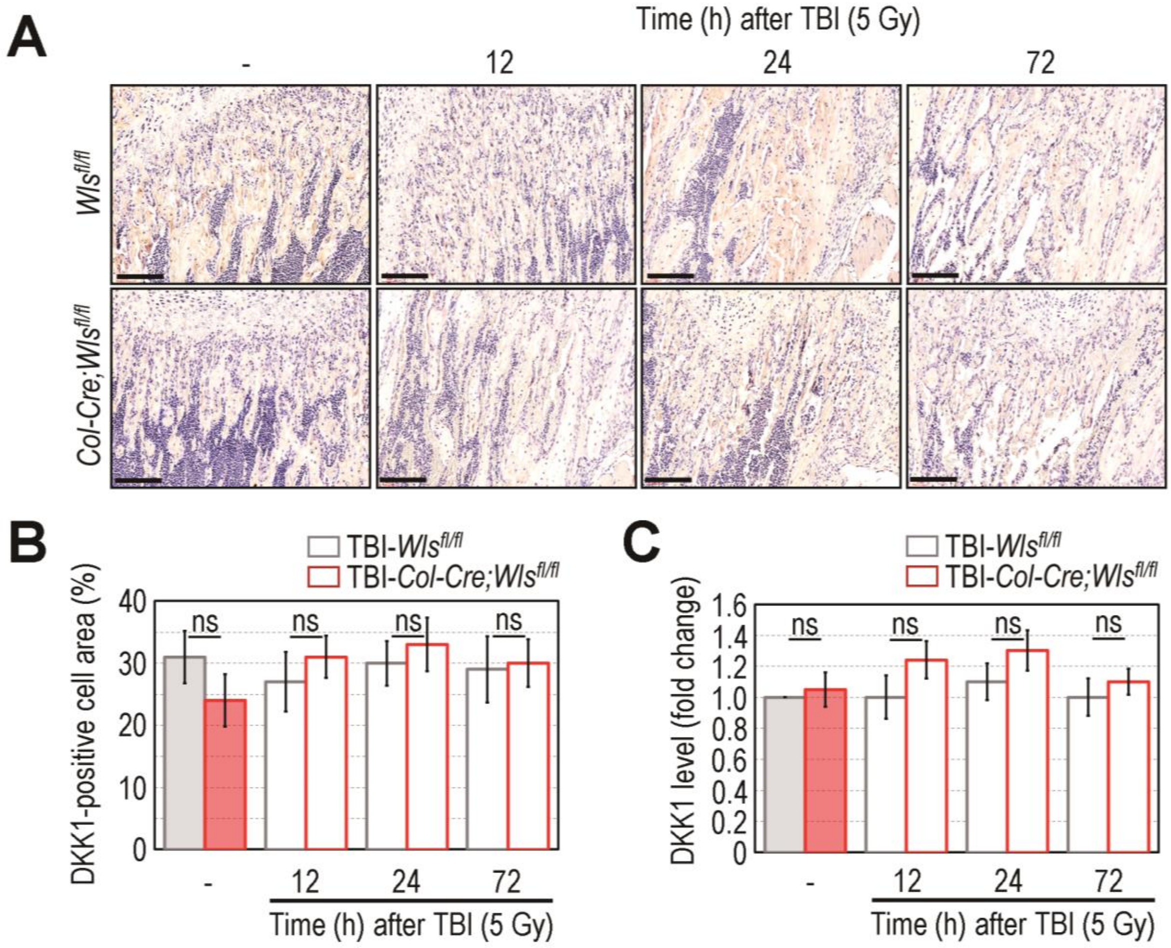

Supplementary Figure 5. TBI does not affect the expression and secretion of DKK1 in the BM of *Wls$^{fl/fl}$* and *Col2.3-Cre;Wls$^{fl/fl}$* mice. **(A)** *Wls$^{fl/fl}$* and *Col2.3-Cre;Wls$^{fl/fl}$* mice at 4 weeks of age were exposed to 5 Gy TBI, and expression pattern of DKK1 in the BM of these mice was evaluated by IHC assay at the indicated times after TBI. Scale bars = 100 µm. **(B)** The area (%) positively stained with DKK1 in IHC assay was calculated (n = 5). **(C)** Protein level of DKK1 in whole BM lysate of the control and mutant mice was determined by ELISA at the indicated times after TBI (n = 5). The significant differences were determined by non-parametric Wilcoxon *t*-test. ns, not significant.

© 2022. Sim HJ et al. Published online at http://www.aginganddisease.org/EN/10.14336/AD.2022.1026

SUPPLEMENTARY DATA

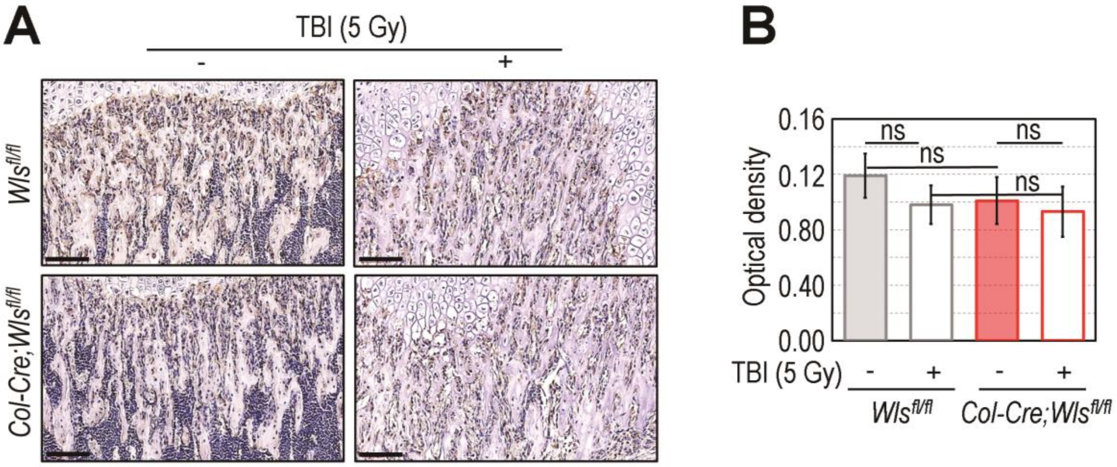

Supplementary Figure 6. The BM level of FGF21 was not changed by osteoblastic *Wls* ablation or in combination with sub-lethal TBI. (A) *Wls$^{fl/fl}$* and *Col2.3-Cre;Wls$^{fl/fl}$* mice at 4 weeks of age were exposed to 5 Gy TBI, and expression level of FGF21 in the BM of these mice was evaluated by IHC assay at 5 days post-TBI. Scale bars = 200 µm. **(B)** The quantified DAB intensity specific to FGF21 in the images was converted to optical density (OD) using the following formula: OD = log (max intensity/mean intensity), where max intensity = 255 for 8-bit images (n = 5). The significant differences were determined by non-parametric Wilcoxon *t*-test. ns, not significant.

© 2022. Sim HJ et al. Published online at http://www.aginganddisease.org/EN/10.14336/AD.2022.1026
